# NF-κB and MicroRNA Deregulation Mediated by HTLV-1 Tax and HBZ

**DOI:** 10.3390/pathogens8040290

**Published:** 2019-12-10

**Authors:** Stefania Fochi, Vincenzo Ciminale, Elisabetta Trabetti, Umberto Bertazzoni, Donna M. D’Agostino, Donato Zipeto, Maria Grazia Romanelli

**Affiliations:** 1 Department of Neurosciences, Biomedicine and Movement Sciences, Section of Biology and Genetics, University of Verona, 37134 Verona, Italy; stefania.fochi@univr.it (S.F.); elisabetta.trabetti@univr.it (E.T.); umberto.bertazzoni@univr.it (U.B.); donato.zipeto@univr.it (D.Z.); 2 Department of Surgery, Oncology and Gastroenterology, University of Padua, 35128 Padua, Italy; v.ciminale@unipd.it; 3 Veneto Institute of Oncology IOV-IRCCS, 35128 Padua, Italy; 4 Department of Biomedical Sciences, University of Padua, 35131 Padua, Italy; dm.dagostino@unipd.it

**Keywords:** HTLV-1, Tax, NF-κB, HBZ, miRNA

## Abstract

The risk of developing adult T-cell leukemia/lymphoma (ATLL) in individuals infected with human T-cell lymphotropic virus 1 (HTLV-1) is about 3–5%. The mechanisms by which the virus triggers this aggressive cancer are still an area of intensive investigation. The viral protein Tax-1, together with additional regulatory proteins, in particular HTLV-1 basic leucine zipper factor (HBZ), are recognized as relevant viral factors required for both viral replication and transformation of infected cells. Tax-1 deregulates several cellular pathways affecting the cell cycle, survival, and proliferation. The effects of Tax-1 on the NF-κB pathway have been thoroughly studied. Recent studies also revealed the impact of Tax-1 and HBZ on microRNA expression. In this review, we summarize the recent progress in understanding the contribution of HTLV-1 Tax- and HBZ-mediated deregulation of NF-κB and the microRNA regulatory network to HTLV-1 pathogenesis.

## 1. Introduction

Hundreds of environmental factors are described as carcinogenic hazards to humans, represented by chemicals, complex chemical mixtures, occupational exposures, physical agents, lifestyle factors, and infectious agents [1]. It has been estimated that approximately 12% of human cancers are caused by viral infections attributed to seven oncoviruses: Epstein-Barr virus (EBV), human herpes virus-8 (HHV-8), human papillomavirus (HPV), hepatitis B and C viruses (HBV and HCV), Merkel cell polyomavirus (MCPyV) and human T-cell lymphotropic virus 1 (HTLV-1) [2,3]. Among them, the most carcinogenic agent is HTLV-1, which causes adult T-cell leukemia/lymphoma (ATLL), a malignancy of mature CD4+ T-cells [4,5,6,7]. HTLV-1 is estimated to infect at least 5–10 million people worldwide, and 3–5% of infected individuals develop ATLL, usually decades after infection [8]. To date, several ATLL therapies have been demonstrated to improve patients’ quality of life, but ATLL prognosis remains poor [9,10]. HTLV-1 is endemic in populations in Southern Japan, the Caribbean, South America, Australia, the Melanesian islands, the Middle East, and in West, Central, and Southern Africa, and it is present with sporadic prevalence in the rest of the world [11,12]. In addition to ATLL, HTLV-1 infection is associated with a severe chronic inflammatory disease of the central nervous system, termed HTLV-1-associated myelopathy/tropical spastic paraparesis (HAM/TSP) [13]. 

Three other viruses closely related to HTLV-1 are named HTLV-2, 3 and 4; among the HTLVs, only HTLV-1 shows a definite link to ATLL development [14]. HTLV-2 has been extensively studied due to its high genomic homology with HTLV-1, but it has a lower pathogenicity [15,16,17]. Comparative studies of HTLV-1 and HTLV-2 have been aimed at identifying peculiar properties of HTLV-1 that may explain its oncogenicity. Cohort studies of HIV-infected persons have indicated that HTLV-2 might be protective against HIV-1 replication, although the role of HTLV-1 and 2 in HIV co-infection remains controversial [18]. 

HTLV-1 oncogenesis is mediated by viral gene products that interact with host proteins and alter their function. The most thoroughly investigated HTLV-1 protein with oncogenic activity is Tax-1, which has the potential to transform rodent fibroblasts, primary human T-cells, and to induce tumors in transgenic mice. Tax-1 is necessary for viral replication and shows no homology with known viral or human proteins [19,20,21]. Tax-1 enhances the expression of genes coded on the plus strand of the proviral genome by recruiting host transcription factors to Tax-responsive elements in the 5′ long terminal repeat (LTR) promoter [22,23]. Through its transactivating activity, Tax-1 deregulates several cell signaling pathways, including NF-κB, AP-1, SRF, and CREB, and thus impinges on the mechanisms controlling cell cycle progression, apoptosis, and the DNA damage response [21,24,25,26]. Both HTLV-1 and HTLV-2 Tax (Tax-1 and Tax-2, respectively) can transform T-cells in vitro [27,28]. Tax-dependent and independent mechanisms of NF-κB activation are considered relevant steps in ATLL development [29,30]. 

Another important oncogenic regulatory protein of HTLV-1, named HTLV-1 basic leucine zipper factor (HBZ), is coded on the minus strand of the provirus [31]. HBZ has been demonstrated to play essential roles in HTLV-1 infectivity and the development of ATLL [25,32,33,34]. The oncogenic potential of HBZ is linked to its ability to induce T-cell proliferation, inhibit cellular senescence, and contribute to viral persistence [35,36,37]. An interesting property of HBZ is its ability to counteract several functions mediated by Tax-1, including activation of the 5’LTR promoter and stimulation of the NF-κB pathway [26,31,32,33,38]. 

Insights into the complexity of HTLV pathogenicity have also been gained through recent investigations of the impact of HTLV-1 infection on the microRNA (miRNA) regulatory network. Several studies identified miRNAs that influence NF-κB signaling, cell proliferation, differentiation, and survival [39,40]. The connections between miRNAs and NF-κB are bidirectional: (i) NF-κB activates the expression of miRNAs that may act as regulators of NF-κB with a negative or positive feedback mechanism; and (ii) miRNAs activated by other cell signaling pathways may modulate NF-κB activity in a crosstalk fashion [41]. 

The present review summarizes recent contributions to the understanding of Tax-1 and HBZ-mediated NF-κB deregulation and the interconnections between NF-κB and the miRNA network that are relevant to HTLV-1-associated cell transformation.

## 2. Tax-Mediated NF-κB Activation

Persistent activation of NF-κB induced by Tax-1 in the early stages of HTLV-1 infection is considered a critical step for survival and proliferation of HTLV-1-infected T cells [30]. The NF-κB transcription factors regulate the expression of more than one hundred genes that participate in cell proliferation, inflammation, and innate immunity [42,43]. HTLV-1 Tax induces constitutive activation of NF-κB, causing aberrant expression of many target genes, including those coding for cytokines/chemokines, inflammatory proteins, adhesion molecules, cell cycle regulators, and apoptosis regulators (representative examples are listed in Table 1) [21,26,44,45,46].

NF-κB is a family of inducible transcription factors represented by five structurally related proteins, named p50, p52, RelA/p65, RelB and c-Rel, which regulate the expression of target genes by binding to κB promoter/enhancer elements as hetero- or homodimers. In the absence of signaling stimuli, the NF-κB proteins are retained in the cytoplasm in association with the inhibitory IκB proteins (IκBα, IκBβ, and IκBε), which mask their nuclear localization signal. Induction of NF-κB is accompanied by the release of NF-κB dimers from IκB, which results in the translocation of NF-κB to the nucleus [65,66]. Activation of NF-κB may occur through the canonical and non-canonical (or alternative) pathways [67]. Activation of the canonical NF-κB pathway is controlled by site-specific phosphorylation and subsequent degradation of IκBα triggered by the IκB kinases complex (IKK). The IKK complex is composed of two catalytic subunits, IKKα and IKKβ, and a regulatory subunit named NF-κB essential modulator (NEMO) or IKKγ [68]. The ubiquitin-dependent degradation of IκBα leads to nuclear translocation of canonical NF-κB factors represented principally by p50/p65 and p50/c-Rel heterodimers [69,70]. Non-canonical NF-κB pathway activation does not involve IκBα degradation, and instead relies on processing of the p100 precursor protein into mature p52. This is triggered by activation of IKKα by NF-κB-inducing kinase (NIK). IKK activation in turn leads to p100 phosphorylation, ubiquitination, and processing, which yields active p52 protein able to form heterodimers with RelB; resulting p52/RelB non-canonical NF-κB complexes translocate into the nucleus and activates target genes [71]. 

Tax-1 activates both the canonical and non-canonical NF-κB pathways through mechanisms that involve interactions with NEMO and the IKK complex [26,72,73,74,75,76,77,78,79,80] and ubiquitination events affecting Tax-1 and pathway components. Indeed, ubiquitination of Tax-1 on C-terminal lysine residues is required for its activation of the NF-κB pathway [81]. Ubiquitination of Tax-1 is catalyzed by the E2 ubiquitin-conjugating enzyme Ubc13 and is required for the ability of Tax-1 to associate with NEMO [73]. Tax-1 also influences ubiquitination by activating E3 ubiquitin ligase-Ring finger protein 8 (RNF8) and Ubc13:Uev1a/Uev2 to assemble Lys63-linked polyubiquitin chains, leading to TAK1 and IKK activation [79]. Conflicting results have been reported on the possible direct role of Tax as an E3 ubiquitin ligase for IKK activation. While Wang et al. suggest that Tax acts as an E3 ubiquitin ligase and Lys63 chains are not essential for Tax-induced IKK activation, Shibata et al. did not confirm Tax-1 E3 ubiquitin ligase activity [80,82], but showed that Tax-1 binds to NEMO and additional components (HOIP and HOIL-1L) of the linear ubiquitin chain assembly complex (LUBAC), forming the LUBAC/Tax-1/IKK complex. Recruitment of the Ubc13/Uev1a E2 complex and unidentified Lys63 E3 enzymes forms an inactive “pre-Taxisome” that allows the generation of Lys63- and linear ubiquitin (Met1 chain)-linked hybrid polyubiquitin chains. This complex represents an active Taxisome that triggers IKK activation through trans-autophosphorylation [82]. Using a proximity-dependent biotinylation approach, Schwob et al. recently demonstrated that p62, a ubiquitin-binding scaffold protein also called sequestosome 1 (SQSTM1), is associated with the Tax/IKK complex and potentiates Tax-mediated NF-κB activation [83]. Two additional factors named Optineurin (OPTN) and Tax1-Binding Protein 1 (TAX1BP1), both belonging to the family of Sequestosome-1 like selective autophagy receptors (SLR), were also shown to enhance Tax-dependent NF-κB activation [84,85]. The interaction of OPTN with Tax in Golgi-associated structures contributes to Tax K63-polyubiquitination in the presence of TAX1BP1 [85]. Further studies are needed to clarify the role of the SRL proteins as well as signaling intermediates, such as TRAF6, RIP1, and MALT1, in Tax-mediated assembly of polyubiquitin chains and to determine whether Tax indeed possesses E3 ubiquitin ligase activity.

Persistent NF-κB hyper-activation promotes cell proliferation and upregulates anti-apoptotic factors such as Bfl-1, Bcl-xL, and survivin [55,56,86], as well as FLICE/caspase-8-inhibitory protein (c-FLIP), an inhibitor of CD95-mediated apoptosis [53,87]. Tax-mediated c-FLIP expression requires both the phosphorylation of IKK and the transcriptional activation of NF-κB [53]. 

Tax-1-induced IKK activation also promotes autophagy through IKK complex-mediated recruitment of Beclin-1 and Bif-1 in lipid draft microdomains [88,89] and augmented formation of autophagosomes [90]. Tax-1 may utilize lipid rafts as a platform to recruit both IKKs and autophagy factors in a unified molecular structure that promotes both NF-κB signaling and autophagy [88]. Tax-1-mediated activation of NF-κB is significantly reduced in Beclin-1-depleted cells, supporting the interpretation that Beclin-1 may modulate NF-κB activity in HTLV-1-infected T cells [89]. The effect of Tax-1 on the deregulation of autophagy has been proposed to promote transformation and survival of HTLV-1-infected T cells [88,89]. 

The cell adhesion molecule 1 (CADM1) may also play a role in Tax-mediated NF-κB deregulation. CADM1 (also known as TSLC1, NECL-2, IGSF4, SynCAM1) acts as a tumor suppressor in a variety of human cancers [91,92] and has been proposed as a cell surface marker for ATLL [93]. Pujari et al. suggest that Tax-1 may require CADM1 to inactivate negative regulators of NF-κB and maintain persistent NF-κB activation [94]. The same authors demonstrated that CADM1 is upregulated by Tax-1 and associates with Tax-1 in cellular complexes containing Ubc13, TAX1BP1, OPTN, and NEMO in membrane lipid raft microdomains [94,95]. However, results of another study of CADM1 expression as a marker of HTLV-1-infected cells did not support a direct role for Tax-1 in its upregulation [96]. The mechanism driving upregulation of CADM1 and its relationship to Tax-1-mediated NF-κB activation thus requires further study, preferably in a cell model that closely recapitulates the natural setting of viral infection.

Tax-1 and Tax-2 share 85% amino acid sequence similarity. Both Tax-1 and Tax-2 contain a CREB-activating domain and zinc finger domains within the N-terminus, two leucine zipper-like motif regions (LZRs), nuclear localization signals (NLSs), a nuclear export sequence (NES), and ATF/CREB-activating domains within the C-terminus [15,97,98,99]. Tax-1 also contains two leucine zipper-like regions that allow the activation of both the canonical and non-canonical NF-κB pathways. In contrast, Tax-2 does not contain these leucine-zipper-like regions and does not activate the non-canonical pathway [100,101]. On the other hand, Tax-2 contains an additional localization domain not present in Tax-1 that contributes to its more pronounced accumulation into the cytoplasm compared to Tax-1 [102]. 

In line with their high functional domain homology, Tax-1 and Tax-2 show interesting similarities in their ability to form complexes with cytoplasmic factors. We demonstrated that both Tax-1 and Tax-2 form complexes with NF-κB pathway components NEMO and p65/RelA, the scaffold protein TAB2, and IKKε and TBK1, two IKK-related kinases that participate in both the NF-κB pathway and signaling pathways mediated by interferon regulatory factors (IRF) [103,104]. We propose that Tax-1 and Tax-2 may be recruited into the TBK1/IKKɛ complexes as scaffolding-adaptor proteins and thereby enhance expression of IFN-inducible genes [104]. Furthermore, we recently demonstrated that TNF Receptor Associated Factor 3 (TRAF3), an inhibitor of the non-canonical NF-κB pathway also acting in IFN-I signaling, interacts with Tax-1 and Tax-2 and is required for efficient NF-κB activation mediated by Tax [105]. 

In HTLV-1-infected and Tax-expressing cells, NF-κB transcription factors may upregulate the expression of target genes that act in a positive feedback loop that maintains the activation of NF-κB. This mechanism has been demonstrated for the transcription factor early growth response factor 1 (EGR1), whose expression and stability are induced by Tax-1-mediated NF-κB activation. Upregulation of EGR1 in turn promotes p65 nuclear translocation and increases NF-κB activation [106].

In apparent contrast with the mitogenic effects of Tax-1 described in many studies, Kuo and Giam provided evidence that chronic hyper-activation of NF-κB induced by Tax-1 triggers cellular senescence [107]. The activation of IKKα and p65/RelA promotes the stabilization of two cyclin-dependent kinase inhibitors, p21 and p27, leading to cell senescence in a p53- and pRb-independent manner [35,107,108,109]. ATLL cells maintain chronic NF-κB activation without undergoing senescence by acquiring genetic/epigenetic changes that mitigate Tax-dependent senescence [110]. It has been proposed that, by modulating NF-κB activity, Beclin-1 may exert a cytoprotective role that favors the escape of Tax-expressing cells from death by driving them toward senescence, thus promoting the survival of infected cells [89]. In line with this model, HTLV-1-transformed T cells show increased levels of constitutive autophagy [88] and reduced viability when autophagic pathways are disrupted, e.g. upon silenging of Beclin-1 [88].

NF-κB activation mediated by Tax-1 may be inhibited by host factors, two interesting examples being CIITA and CYLD. CIITA (MHC class II transactivator) favors the retention of the inactive p50/RelA/IκB complex in the cytoplasm, thus blocking Tax-dependent activation of NF-κB-responsive genes [111]. This effect was proposed to represent a host cell response that impedes virus spread, viral replication, and Tax-1-dependent cell transformation. Wu et al. demonstrated that interaction of Tax-1 with the de-ubiquitinase CYLD leads to Taxν1’s deubiquitination with consequent loss of Tax-stimulated IKK activation [112]. They also observed that Tax-1-expressing cells show an increase in CYLD phosphorylation, a modification that interferes with its enzymatic activity; Tax-induced phosphorylation of CYLD may thus represent a feedback mechanism that hinders host cell defences to viral infection [112].

## 3. Deregulation of NF-κB Activation by HBZ

While Tax-1 expression is frequently lost in ATLL cells, HBZ remains detectable in virtually all ATLL samples. HBZ is constitutively expressed in all ATLL cases, whereas Tax-1 is detected in only about 30% of ATLL cells. HBZ attenuates the effects of Tax-1 on NF-κB activation and senescence, thus contributing to viral latency and persistence [32,35,113]. Analyses of HTLV-1 HBZ-mutant viruses demonstrated that HBZ is dispensable for viral replication and immortalization of primary human T cells [114]. However, both Tax-1 and HBZ are essential for ATLL development, with Tax-1 necessary for T-cell transformation in the initial steps of tumorigenesis and HBZ required for maintaining the transformed phenotype [115,116]. Tax-1-mediated activation of NF-κB represents an essential step to promote cell proliferation, which however must be balanced by HBZ to allow infected cells to escape immune surveillance. In ATLL cells, NF-κB is persistently activated even in the absence of Tax-1 due to mutations and epigenetic alterations affecting genes involved in the NF-κB pathway and its regulation [29].

HBZ suppresses the transcription of various NF-κB target genes, including IL-8, IL2RA, IRF4, VCAM-1, CCND1, and VEGF [26,32,117,118]. The molecular mechanism of HBZ-mediated NF-κB inhibition has been investigated in several studies, which revealed that HBZ selectively suppresses the canonical NF-κB pathway by (i) interacting with p65 and reducing its DNA binding capacity, (ii) increasing the expression of PDLIM2, an E3 ubiquitin ligase that determines p65 degradation, and (iii) reducing p65 acetylation [117,119,120]. 

Comparative studies have highlighted differences between HBZ and its HTLV-2 homologue APH-2 which may contribute to determining the different pathogenicities of the two viruses [121,122]. APH-2 is dispensable for viral infection and transformation [121]. Both HBZ and APH-2 counteract the activation of the NF-κB by Tax, but they differ in the mechanisms of inhibition [26,105]. Unlike APH-2, HBZ does not interact with Tax-1, whereas APH-2 does not drastically decrease p65 expression or induce its ubiquitination [119]. We demonstrated that HBZ and APH-2 also differ in their intracellular localization and that only APH-2 forms complexes with cytoplasmic host factors and Tax-2 [105]. We also found that APH-2 is more effective than HBZ in inhibiting NF-κB activation by Tax, and we proposed a model in which the recruitment of APH-2 in cytoplasmic structures with NF-κB factors may inhibit p65’s nuclear translocation, leading to NF-κB inhibition [105]. Another property of APH-2 that distinguishes it from HBZ is its targeting to PML nuclear bodies, which leads to proteasomal degradation of APH-2 in a SUMO-dependent manner [123]. 

## 4. Deregulation of miRNA Expression by Tax-1 and HBZ 

miRNAs are a class of small non-coding RNAs of ~22 nucleotides that can inhibit mRNA translation or degrade target transcripts upon binding to their untranslated regions (UTRs) through imperfect base-pairing [124]. Most miRNAs are transcribed by RNA polymerase II from intergenic, intronic, or polycistronic loci as long primary miRNA transcripts (pri-miRNA), which are successively processed to yield a smaller stem-loop (pre-miRNA, approximately 70 nt) by a nuclear complex containing an RNA binding protein, DGCR8, and an endoribonuclease, DROSHA. The pre-miRNA is then exported from the nucleus to the cytoplasm and cleaved by the endoribonuclease DICER, which removes the terminal loop to produce a mature miRNA duplex. One strand of the duplex combines with Argonaute (AGO) proteins into the RNA-induced silencing complex (RISC) [125,126,127].

Several studies revealed alterations in the expression of miRNAs in HTLV-1-infected cell lines, ATLL cell lines, and ATLL samples, and highlighted the important role of miRNA deregulation in ATLL pathogenesis [128,129,130,131,132,133,134,135]. A global profiling analysis of miRNA expression in a large panel of ATLL samples indicated a trend toward downregulation of miRNA expression, with particularly conspicuous repression of miR-31 [135]. This study identified NIK, a positive regulator of the non-canonical NF-κB pathway, as an important target of miR-31; Polycomb-mediated epigenetic silencing of miR-31 causes aberrant overexpression of NIK in ATLL cells, thus contributing to NF-κB activation [135]. 

Studies aimed at understanding the impact of individual viral proteins on the miRNA network identified several miRNAs whose expression is influenced by Tax-1 and HBZ (Table 2).

**Figure 1:**
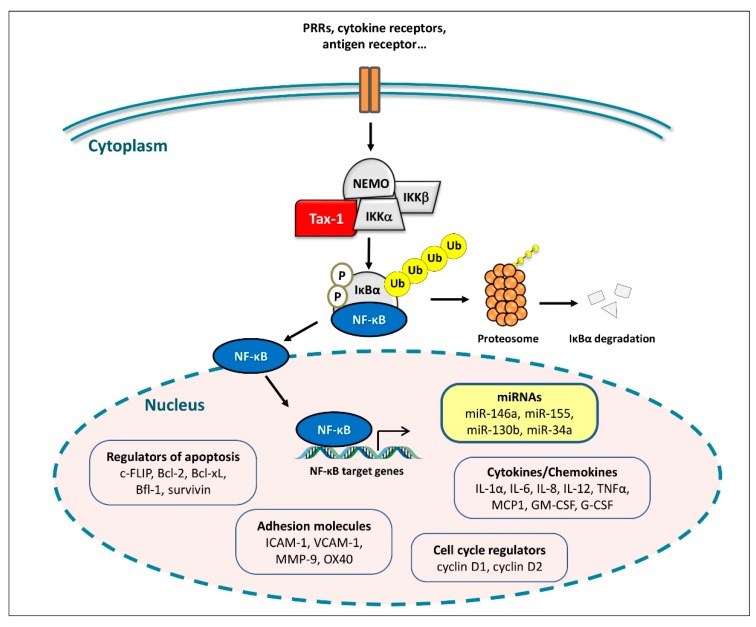
NF-κB target genes up-regulated by Tax-1-mediated NF-κB activation. Interaction of Tax-1 with the IκB kinases (IKK) complex leads to degradation of IκBα by the proteasome and nuclear translocation of active NF-κB. NF-κB activates transcription of genes involved in the production of inflammatory cytokines, adhesion molecules, and regulation of apoptosis and the cell cycle. Four selected miRNAs (miR-146a, miR-155, miR-130b and miR-34a) are demonstrated to be upregulated by Tax-1-mediated NF-κB activation (as described in the text).

Several miRNAs are positively regulated by NF-κB and are thus expected to be over-expressed in the context of Tax-mediated NF-κB hyperactivation. These include miR-146a, miR-155, miR-130b, and miR-34a, all of which contain binding sites for NF-κB in their promoters (Figure 1). 

miR-146a plays a crucial role in regulating the proliferation of immune cells and inhibiting the inflammatory response [142]. miR-146a is up-regulated in HTLV-1-infected T-cell lines and is induced by Tax-1 in an NF-κB-dependent manner [129]. Tax-mediated induction of miR-146a was shown to enhance the growth of an HTLV-1-infected T-cell line [137]. On the other hand, studies performed in breast cancer cell lines showed that miR-146a acts as a negative regulator of constitutive NF-κB activity by targeting TRAF6 [143], suggesting that miR-146a may act on NF-κB in a negative feedback loop in this cell context. This mechanism of regulation seems to be ineffective in HTLV-1-infected cells, which maintain persistent activation of NF-κB. Interactions between miR-146a and additional target mRNAs identified in other cell systems, including IRAK 1 (interleukin 1 receptor associated kinase 1), IL-8, IL-6, the chemokine receptor CXCR4, and matrix metalloproteinase-9 [143,144] might also play a role in HTLV-1 infected cells.

miR-155 participates in the regulation of the immune response and is involved in maturation and differentiation of lymphocytes [145]. Similar to its effects on miR-146a, Tax-1 was shown to induce expression of miR-155 via the NF-κB and AP-1 signaling pathways; inhibition of miR-155 with an anti-miR-155 oligonucleotide was shown to reduce the growth of HTLV-1-positive T-cell lines [139]. miR-155 targets described in other cell systems whose downregulation might play a role in HTLV-1 infection include tumor protein 53-induced nuclear protein 1 (TP53INP1), which promotes cell cycle arrest and apoptosis, the transcriptional repressor BACH1, and human immunodeficiency virus type 1 enhancer binding protein 2 (HIVEP2), which inhibits the expression of c-Myc [146,147].

The miR-130b gene contains several transcription factor binding sites that are Tax-responsive, including sites for NF-κB, and its expression was confirmed to be upregulated by Tax-1 in transfected cells [128]. miR-130b in turn regulates expression of the tumor suppressor protein TP53INP1, thus supporting a role for this miRNA in favoring the proliferation and survival of HTLV-infected cells [128].

miR-34a is a component of the p53 pathway; in addition to p53, its expression is transcriptionally regulated by NF-κB and other transcription factors [148,149,150]. miR-34a targets include transcripts coding for proteins that control cell proliferation and survival, and its expression in most normal cell types promotes growth arrest, senescence, or apoptosis [151]. While miR-34a is downregulated in many cancer types [152,153,154], we observed that miR-34a is more abundant in HTLV-1-infected cell lines and in ATLL cells compared to control CD4+ cells [136,155], and provided evidence that both p53 and NF-κB promote miR-34a expression in the context of HTLV-1 infection [136]. Experiments performed using a miR-34a ‘sponge’ construct and a synthetic miR-34 mimic provided evidence that abundant miR-34a levels provide a survival advantage to HTLV-1-infected cells, in part by controlling the expression of the pro-apoptotic BCL2 family member Bax [136]. However, forced expression of Tax-1 in HeLa cells resulted in only marginal upregulation of miR-34a through a mechanism that did not appear to depend on Tax-1’s ability to stimulate NF-κB [136]; the possible direct influence of Tax-1 on miR-34a expression in the context of infected cells remains to be investigated.

Selected miRNAs are downregulated by Tax-1 or HBZ. Rahman et al. (2012) found that at least four miRNAs, including miR-135b, miR-149, miR-872, and miR-873, are significantly downregulated in Jurkat cells ectopically expressing Tax-1 [138]. Two of them, miR-149 and miR-873, regulate the expression of the chromatin remodeling enzymes p/CAF and p300, suggesting a role for Tax-1 in regulating the expression of histone acetyltransferase (HAT) family factors [138]. Vernin et al. (2014) proposed that an HBZ/miRNA axis may induce genomic instability [141]. They showed that HBZ increases the expression of miR-17 and miR-21 which in turn suppress the expression of nucleic acid binding protein 1 (NABP1, also known as OBFC2A), a participant in DNA repair [141].

### Deregulation of DICER and DROSHA Mediated by Tax-1 and HBZ

**Figure 2:**
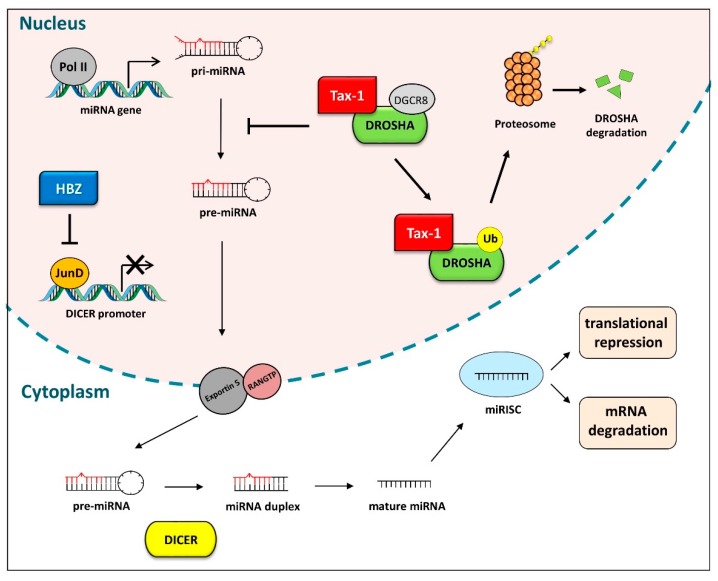
Tax-1 and HBZ interfere with miRNA processing. Within the nucleus, most miRNAs are synthesized as pri-miRNAs by RNA polymerase II and processed into pre-miRNAs by DROSHA/DGCR8-containing complexes. Pre-miRNAs are exported into the cytoplasm by Exportin 5, where they undergo an additional processing event by DICER, generating a miRNA duplex. The formation of the RNA-induced silencing complex (RISC) complex leads to repression of translation and/or degradation of specific target transcripts. Tax-1 deregulates this process by directly interacting with DROSHA and leading to its proteasomal degradation (the nuclear or cytoplasmic location of this process remains to be determined). HBZ reduces the expression of DICER by removing JunD from the binding sites located in the DICER gene promoter.

As mentioned above, miRNA profile analyses of ATLL cells revealed a relevant decrease in expression of many miRNAs [135]. This may in part reflect the ability of Tax-1 and HBZ to interfere with the cellular miRNA processing machinery (Figure 2). Van Duyne et al. (2012) reported that DROSHA protein expression is downregulated in primary HTLV-1-infected cells, HTLV-1-infected cell lines and HTLV-1-transfected cells [156]. They proposed that HTLV-1 may deregulate the cellular miRNA machinery by promoting degradation of DROSHA, thus repressing its functionality. Further analyses revealed that Tax-1 forms complexes with DROSHA in the nucleus, which leads to degradation of DROSHA by the proteasomal pathway. Analyses of HTLV-1-infected cells indicated that the loss of DROSHA and DGCR8 increases viral replication, suggesting that such interference with the RNAi pathway may confer an advantage to the virus [156].

HBZ also participates in the deregulation of host miRNAs. A recent study demonstrated a decrease in the abundance of miR-Let7-a, miR-16, miR-20, miR-21, miR-31, miR-93, miR-125a, miR-132, miR-143, miR-155, miR-200, and miR-873 in HBZ-expressing T cells and ATLL cells [140]. This reduction was attributed to the downregulation of DICER expression due to HBZ-mediated removal of JunD from the AP-1 binding sites located in the DICER promoter. Interestingly, Valproate, a histone deacetylase inhibitor (HDACi) used in cancer therapy, was found to restore DICER expression and miRNA maturation in HTLV-1-infected cells [140]. Studies of the HTLV-1 post-transcriptional regulatory protein Rex indicate that it also interferes with host miRNA maturation by inhibiting DICER’s enzymatic activity [157]. The alterations in miRNA expression in HTLV-1-infected cells are therefore complex and presumably depend on the balance between the strength of positive effects of Tax-1 and HBZ on the expression of specific miRNAs and their capacity for inducing a general downregulation of miRNA processing.

## 5. Conclusions

Nearly forty years of research dedicated to understanding the link between HTLV-1 and ATLL have consolidated the crucial roles of Tax-1 and HBZ in altering multiple pathways that govern cell proliferation, senescence and death, with the deregulation of NF-κB representing a hub, and miRNAs acting as fine-tuners of target gene expression. After infection, Tax-1-dependent NF-κB signaling activation plays a pivotal role in the proliferation and initial polyclonal expansion of HTLV-1-infected T cells. An ensuing balance between the effects of Tax-1 and HBZ on NF-κB activation contributes to the escape of infected cells from the host innate immune response while promoting cell survival and proliferation. The crosstalk between NF-κB and autophagy may also promote T cell survival and malignant transformation. Despite abundant information on the molecular mechanisms that lead to the deregulation of the NF-κB pathway by Tax-1, several areas of investigation still pose important questions. How does NF-κB activation contribute to chemoresistance in the aggressive forms of ATLL? Do miRNAs upregulated by Tax-1 contribute to autophagosome accumulation? What are the mechanisms that allow Tax-1 and HBZ to fine tune cell senescence while ensuring clonal expansion of infected cells? May autophagy be a potential therapeutic target in ATLL treatment? Given the central role of NF-κB signaling in the proliferation and survival of infected cells, further understanding of the interconnections between the viral regulatory proteins acting on the NF-κB and miRNA networks will likely offer opportunities for the development of novel pharmacological approaches and personalized treatments to eradicate HTLV-1 infection and cure ATLL. 

## Figures and Tables

**Table 1:** Examples of NF-κB-dependent target genes whose expression is deregulated by Tax.

Gene Symbol	Gene Description	Effect	Reference
**Cytokines/Chemokines**			
IL1A	Interleukin 1 alpha	Upregulation	[47]
IL6	Interleukin 6	Upregulation	[48]
CXCL8	Interleukin 8	Upregulation	[49]
IL12B	Interleukin 12	Upregulation	[50]
TNF	Tumor necrosis factor-alpha	Upregulation	[50]
MCP1	Monocyte chemoattractant protein 1	Upregulation	[50]
CSF2	Granulocyte-macrophage colony stimulating factor	Upregulation	[51]
CSF3	Granulocyte-colony stimulating factor	Upregulation	[51]
**Cell Cycle Regulators**			
CCND1	Cyclin D1	Upregulation of cyclins D1 and D2 by Tax is involved in IL-2–independent growth of mouse T cells.	[52]
CCND2	Cyclin D2		[52]
**Regulators of Apoptosis**			
CFLAR	FLICE/Caspase-8-inhibitory protein	Upregulation of c-FLIP by Tax inhibits Fas-mediated apoptosis in HTLV-1-infected T cells.	[53]
BCL2	Bcl-2 apoptosis regulator	Upregulation of the antiapoptotic protein Bcl-2 is mediated by the co-expression of Tax and p21^WAF1^.	[54]
BCL2L1	Bcl-2-like long	Upregulation	[55]
BCL2A1	BCL2 related protein A1	Upregulation of anti-apoptotic Bfl-1 protein by Tax contributes to survival of HTLV-1-infected T cells.	[56]
BIRC5	Baculoviral IAP repeat containing 5/survivin	Upregulation of survivin participates in Tax-mediated resistance to apoptosis.	[57]
**Adhesion Molecules**			
ICAM1	Intercellular adhesion molecule 1	Upregulation	[58]
VCAM1	Vascular cell adhesion molecule 1	Upregulation	[59]
MMP9	Matrix metallopeptidase 9	Upregulation	[60]
TNFRSF4	OX40 cell surface antigen	Upregulation	[61]
**NF-κB Negative Regulators**			
TNFAIP3	TNF alpha induced protein 3/A20	Upregulation Tax inactivates A20 function disrupting the interaction with TAX1BP1 and Itch.	[62,63]
IKBKB	Inhibitor of nuclear factor kappa B kinase beta	Downregulation. Tax induces the degradation of IκBβ.	[64]

**Table 2:** Deregulated host miRNAs in Tax- or HBZ-expressing cells.

miRNA	miRNA Expression	Effect	Reference
**Tax-Expressing Cells**			
miR-34a	up	n.d.	[136]
miR-146a	up	Cell growth and proliferation	[129,137]
miR-149	down	Chromatin remodeling	[138]
miR-130b	up	Cell proliferation and survival	[128]
miR-135b	down	Chromatin remodeling	[138]
miR-155	up	Cell growth	[139]
miR-872	down	Chromatin remodeling	[138]
miR-873	down	Chromatin remodeling	[138]
**HBZ-Expressing Cells**			
miR-Let7-a	down	n.d.	[140]
miR-16	down	n.d	[140]
miR-17	up	Genetic instability and cell proliferation	[141]
miR-20	down	n.d.	[140]
miR-21	up	Genetic instability and cell proliferation	[141]
miR-31	down	n.d.	[140]
miR-93	down	n.d.	[140]
miR-125a	down	n.d.	[140]
miR-132	down	n.d.	[140]
miR-143	down	n.d.	[140]
miR-155	down	n.d.	[140]
miR-200	down	n.d.	[140]
miR-873	down	n.d.	[140]

n.d. = not determined.
